# The complete chloroplast genome of *Prunus apetala*, a native deciduous flowering cherry of Japan

**DOI:** 10.1080/23802359.2019.1673226

**Published:** 2019-10-04

**Authors:** Xin Shen, Xinhong Liu, Wenxin Li, Dongyue Jiang

**Affiliations:** Zhejiang Academy of Forestry, Hangzhou, Zhejiang, P. R. China

**Keywords:** *Prunus apetala*, chloroplast genome, pair-end sequencing, phylogenetic position

## Abstract

The complete chloroplast (cp) genome of *Prunus apetala* was determined. The length of the cp genome of *P. apetala* is 157,987 bp. A total of 129 genes are comprised of 84 protein-coding genes, 37 tRNA genes, and 8 rRNA ribosomal genes. The overall GC content of cp genome is 36.7%. The phylogenetic position of *P. apetala* is sister to *Prunus serrulata* var. *spontanea*.

*Prunus apetala* (Rosaceae) is a native species of cherry to Japan, endemic to Honshu. This species is also called clove cherry for its clove-shaped calyx. It is a deciduous small tree or shrub with the height of 4–7 m. The petal is pure white or with pink shade, and fall very soon (Iwatsuki et al. 2001). Here, we assembled the chloroplast (cp) genome of *P. apetala* (MN242945) and analyzed its phylogenetic status in Amygdaleae, which will be helpful for future studies on the conservation, phylogeny, and breeding of *Cerasus*.

The fresh leaves of *P. apetala* were collected from Tama forest science garden, Tokyo, Japan (35°38'45.5''N 139°16'46.2''E). A voucher specimen (JDYY070) was deposited in the Herbarium of Zhejiang Academy of Forestry, Hangzhou, China. The genomic DNA was extracted from a single individual of *P. apetala* using Hi-DNAsecure Plant Kit (Tiangen, Beijing, China), and used to generate a short-insert (<800 bp) paired-end sequencing library. Paired-end sequencing was conducted on HiSeq XTM Ten analyzer (Illumina, San Diego, CA) at Beijing Genomics Institute (BGI, Shenzhen, China). The chloroplast DNA sequences were manually confirmed using Geneious (R10.2.3) with *Prunus maximowiczii* (KP760071) as a reference, and assembled by SPAdes (Bankevich et al. 2012; Kearse et al. 2012). The cp genome annotation and correction were performed using Plann and Sequin (Huang and Cronk 2015).

The size of chloroplast genome of *P. apetala* is 157,987 bp in length with 36.7% GC content, including a pair of inverted repeat (IR, 26,436 bp) regions, a small single copy (SSC, 19,121 bp) region, and a large single copy (LSC, 85,994 bp) region. A total of 129 genes were successfully annotated, containing 84 protein-coding genes (PCG), 37 tRNA genes, 8 rRNA ribosomal genes. Most of genes occur as a single copy, while 6 PCGs, 7 tRNA genes, and 4 rRNA genes in IR regions are duplicated.

To confirm phylogenetic position of *P. apetala*, the complete cp genome sequences of 19 Amygdaleae species were used to construct the phylogenetic tree. The maximum-likelihood (ML) was performed using PhyloSuite with 1000 bootstrap replicates (Zhang et al. 2018). The phylogenetic analysis showed that all the species are divided into three clades, subg. *Padus*, subg. *Prunus*, and subg. *Cerasus*, similar to the previous results (Kim et al. 2019), and *P. apetala* is sister to *Prunus serrulata* var. *spontanea* (Figure 1).

**Figure 1.**
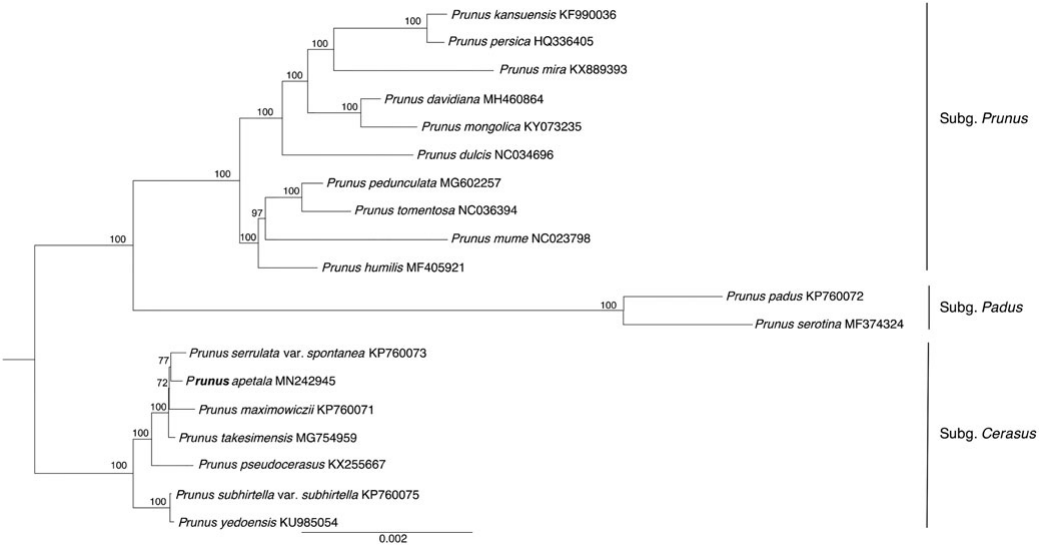
Maximum-likelihood phylogenetic tree for *Prunus apetala* based on 19 complete cp genomes with 1000 bootstrap replicates.

## References

[CIT0001] Bankevich A, Nurk S, Antipov D, Gurevich AA, Dvorkin M, Kulikov AS, Lesin VM, Nikolenko SI, Pham S, Prjibelski AD, et al. 2012 SPAdes: a new genome assembly algorithm and its applications to single-cell sequencing. J Comput Biol. 19:455–477. 22506599 10.1089/cmb.2012.0021 PMC3342519

[CIT0002] Huang DI, Cronk Q 2015 Plann: a command-line application for annotating plastome sequences. Appl Plant Sci. 3:1500026. 10.3732/apps.1500026 PMC4542940 26312193

[CIT0003] Iwatsuki K, Boufford DE, Ohba H 2001 Flora of Japan: Angiospermae-Dicotyledoneae: Archichlamydeae. Tokyo (Japan): Kodansha; p. 136–137.

[CIT0004] Kearse M, Moir R, Wilson A, Stones-Havas S, Cheung M, Sturrock S, Buxton S, Cooper A, Markowitz S, Duran C, et al. 2012 Geneious Basic: an integrated and extendable desktop software platform for the organization and analysis of sequence data. Bioinformatics. 28:1647–1649. 22543367 10.1093/bioinformatics/bts199 PMC3371832

[CIT0005] Kim HT, Kim JS, Lee YM, Mun JH, Kim JH 2019 Molecular markers for phylogenetic applications derived from comparative plastome analysis of *Prunus* species. J Sytemat Evol. 57:15–22.

[CIT0006] Zhang D, Gao F, Li WX, Jakovlić I, Zou H, Zhang J, Wang GT 2018 PhyloSuite: an integrated and scalable desktop platform for streamlined molecular sequence data management and evolutionary phylogenetics studies. bioRxiv. doi:10.1101/489088 31599058

